# Notes and Comments

**Published:** 1899-07

**Authors:** 


### Notes and Comments.[1]

Independent Journalism.—In speaking upon the subject of independent journalism, Dr. Charles A. Brackett referred to the International Dental Journal in the following manner:

"The International Dental Journal has always had high merit in its reports of society meetings and in its extended original articles. It has been said that it might do better in having, in addition, more short, simple items not requiring much energy to write or to read, but which would be practical suggestions. It is in the power of every dentist to make, within the compass of a few lines, a half-page, more or less, reports of cases, suggestions about doing certain things and overcoming difficulties, that would be useful to others. If we will, each one of us, two or three times a year, contribute ten lines to the Journal, the aggregate will be a large mass of material that will increase its attractiveness and be a great benefit to all concerned."

It was for just such a purpose that the department of "Notes and Comments" was created, but too few have contributed to it. We hope many will follow the suggestion of Dr. Brackett, which is also given in the foot-note below, of long standing, and assist in advancing the usefulness of this department.

---

Does License to practise Medicine permit the Practice of Oral Surgery?—A curious case for medico-legal decision, reported in the *Dominion Dental Journal*, has arisen in the State of Rhode Island. Registration for medical practice is granted in

[1] The assistant editor solicits contributions for this department,—new methods, new remedies and formulas, or any short practical note which may prove of value to the practitioner or student. Address 1338 Walnut Street, Philadelphia.

Rhode Island upon application to the State Board of Health by graduates of recognized medical colleges, while dentists are required, by a recent enactment, to pass an examination before the State Board of Registration in Dentistry. A graduate in dental surgery and in medicine, after having been registered by the State Board of Health, and securing his license, engaged in the practice of medicine, including dental and oral surgery. He was forthwith arrested for infraction of the law requiring examination and license for the practice of dental surgery, and the case now awaits judicial decision. Logically, it would be reasoned that one qualified to practise medicine and so authorized to do by State authority, would at the same time be conceded the qualification and the license to practise any part of medicine, ophthalmology, otology, obstetrics, gynæcology, oral and dental surgery, etc. To ask a qualified and licensed medical man to pass a special examination in oral and dental surgery would not be more ridiculous than to ask him to pass a special examination and secure a special license for the practice of midwifery. It would seem, further, from the legal point of view, that when there is conflict in letter between existing legislation the spirit must be followed, and surely no one would contend that the part is greater than the whole. The question seems a very simple one, and the solution should be attended with no difficulty or complication. It all revolves about the point whether an approved graduate in medicine shall be permitted to practise all branches of it.

---

Dental Knowledge in the Medical Profession.—In writing upon this subject, Professor Fillebrown reports a case where an eminent surgeon took a man suffering from an abscess caused by the root of an inferior molar—and only half a root at that—and operated on him twice for necrosis, leaving big scars on the jaw. That patient finally came to what medical men regard as a second-rate institution—a dental infirmary—and received complete relief. So long as such ignorance prevails we cannot expect medical men to understand the position of dentistry. If our medical schools will insist that physicians shall know as much about the relations of the teeth to the various parts of the human system and the influence of their diseased conditions as they do about the effect of other diseased organs on the body, we shall then have a physician who will respect dentistry, and who will be a great deal more of a man himself. So long as our medical schools ignore a part of the human system, and neglect to instruct their students as to what its relations are, just so long will this unsatisfactory state of affairs exist.

---

Do we wish to be Medical Specialists?—Dr. J. L. Williams, in a paper before the New York Odontological Society, says, "We have been hammering and clamoring for I know not how many years at the door of Medicine, asking, beseeching, begging for recognition as medical specialists; we give ourselves all sorts of names, oral surgeons, oral specialists, stomatologists, and what not, in the hope that we may gain a nod of recognition from the world that shall imply we are something more than what is generally conveyed by the term "dentist;" we have done nearly everything except the right thing, the dignified, manly thing. Now, if we will go to work in real honest earnest, we shall soon enter into our rightful and desired position without asking permission of any of the older organizations. The man who feels that he is master of the situation asks no favors of anybody."

---

Lead-Tin and Lead-Antimony Alloys, quick testing.—Mr. Joseph Richards, of Philadelphia, describes in the *Journal of the Franklin Institute*, May, 1899, page 398, a ready and quick method of testing the above alloys, which he has invented and reduced to commercial use. The bulk of lead-ores are contaminated with antimony, and in the refining of lead the antimony, when at a dull red heat, is rapidly oxidized, and floats on the top of the molten metal as a scum. This is removed as fast as formed, and in the course of from ten to forty-eight hours all the antimony is eliminated. It is desirable in this process that the workman should know how he is progressing with his work. In order to meet this demand, Mr. Richards made a series of tests of the relative weights of standard alloys of antimony and lead, from pure lead up to twenty-four per cent. of antimony. (Lead becomes saturated with twenty-four per cent. of antimony, no more will unite with it, and any in excess of this floats upon the molten mass; so that this is the limit to which this method of testing can be applied.) Castings were made of these alloys, and the average weight of each carefully ascertained. With this data, he constructed a balance so graduated that when balanced with a bullet of alloy cast in a standard mould in the pan, the tee marked the exact percentage of antimony in the alloy.

During his experiments he noticed a peculiar behavior of some of these alloys. While antimony is lighter than lead, he found that an alloy containing less than two per cent. of antimony weighed more than pure lead. To meet this difficulty he took advantage of the physical property of these alloys forming, when a small button is poured upon a flat plate, a surface not at all like lead, but of a fine, white, crystalline appearance. So he has supplemented his testing balance with a set of buttons of a known composition, from two per cent. down to zero, changing composition by one-tenth of one per cent. Thus the operator can go on with his work until the button shows a perfectly lustrous surface free from crystals; at this point the lead will be 998 fine or over.

He uses a similar process for testing lead-tin alloys, and also for testing what is commercially known as terne plates, sheets of iron coated with an alloy of lead and tin, used for roofing purposes. The balances are so constructed for each of these uses that no calculation is necessary, the position of the tee gives at a glance the information desired to a quarter of one per cent. To make the same test by the usual chemical process requires several hours.

---

Death from Attempt at Tooth-Extraction.—The following case is reported by the *Dental Record*, of London, England. A young man, eighteen years of age, endeavored to extract the roots of a left upper molar by means of a sharpened wooden penholder. After these attempts at amateur tooth-extraction he suffered severe pain in the neighborhood of the roots and some swelling, which increased so as to involve the cheek adjacent to it. Three days later a blackish slough commenced to form and the patient applied for treatment to the Dental Hospital of London, where he was ordered a mouth-wash. Two days later he again went to the hospital, and, when seen by the surgeon, was found to have a blackish slough, separated by a distinct line of demarcation from the surrounding tissues, which were much inflamed. The slough occupied the region of the upper left molar teeth and extended inward into the palate, involving it to a considerable extent. The temperature was 102.6° F.; pulse-rate, 120; the tongue was foul and the patient looked ill. The patient was sent to Charing Cross Hospital, where he was admitted, and having been anæsthetized the slough was freely scraped away and the tissues cauterized with pure carbolic. In spite of this treatment another slough formed, the adjacent teeth became loose, and the bone was stripped and laid bare by burrowing pus, which could be freely squeezed out. The patient was again anæsthetized and the parts were again freely scraped. Owing to great respiratory embarrassment during the anæsthesia trachetomy was performed. The edges of this wound also showed signs of sloughing, and gradually the patient sank and died.

